# Exogenous ATP Attenuates Biochemical and Histological Markers of Osimertinib-Associated Myocardial Injury in Rats

**DOI:** 10.3390/antiox15101227

**Published:** 2026-09-23

**Authors:** Engin Hendem, Esra Tuba Sezgin, Bahadir Suleyman, Renad Mammadov, Sevinc Haciyeva, Mustafa Ozkaraca, Taha Abdulkadir Coban, Halis Suleyman

**Affiliations:** 1 Department of Medical Oncology, Mengucek Gazi Education and Research Hospital, Erzincan Binali Yıldırım University, Erzincan 24100, Turkey; enginhende61@gmail.com; 2 Anesthesia Program, Vocational School of Health Services, Erzincan Binali Yıldırım University, Erzincan 24036, Turkey; esra.demir@erzincan.edu.tr; 3 Department of Medical Pharmacology, Faculty of Medicine, Erzincan Binali Yıldırım University, Erzincan 24100, Turkey; bsuleyman@erzincan.edu.tr (B.S.); rmammadov@erzincan.edu.tr (R.M.); 4 Department of Pathological Physiology, Azerbaijan Medical University, Baku AZ1000, Azerbaijan; sevinchaciyeva890@gmail.com; 5 Department of Pathology, Faculty of Veterinary Medicine, Sivas Cumhuriyet University, Sivas 58140, Turkey; mustafaozkaraca@cumhuriyet.edu.tr; 6 Department of Medical Biochemistry, Faculty of Medicine, Erzincan Binali Yıldırım University, Erzincan 24100, Turkey; acoban@erzincan.edu.tr

**Keywords:** osimertinib, ATP, cardiotoxicity, oxidative stress, antioxidant defense, cardiac injury, 8-OHdG, troponin I

## Abstract

(1) Background: Osimertinib is an effective third-generation EGFR tyrosine kinase inhibitor, but its clinical use may be limited by cardiotoxicity. This study investigated the relationship between reduced measured cardiac ATP levels and osimertinib-associated cardiac injury and examined whether exogenous ATP administration attenuates the associated biochemical and histopathological alterations. (2) Methods: Twenty-four male Wistar rats were randomly allocated to healthy control (HG), osimertinib (OSM), ATP 5 mg/kg + osimertinib (AOSM-5), and ATP 10 mg/kg + osimertinib (AOSM-10) groups (*n* = 6). ATP was administered intraperitoneally once daily, followed 1 h later by osimertinib (50 mg/kg, oral gavage) for three weeks. Cardiac tissue ATP, MDA, and tGSH levels, SOD and CAT activities, and plasma TPI levels were measured, together with histopathological and immunohistochemical evaluation of COX-1, COX-2, and 8-OHdG. (3) Results: Osimertinib significantly decreased ATP and tGSH levels and SOD and CAT activities, and increased MDA and TPI levels (*p* < 0.001), accompanied by increased COX-2 and 8-OHdG, reduced COX-1, hemorrhage, and mononuclear cell infiltration. Exogenous ATP attenuated these alterations, with generally greater effects observed at 10 mg/kg. (4) Conclusions: These findings indicate that ATP co-administration attenuated biochemical, histopathological, and immunohistochemical alterations associated with osimertinib-induced myocardial injury in rats, with these effects being associated, at least in part, with reduced oxidative stress. However, the functional cardiac effects, underlying mechanisms, and potential influence of ATP administration on the antitumor efficacy of osimertinib require further investigation.

## 1. Introduction

Osimertinib is a third-generation epidermal growth factor receptor (EGFR) tyrosine kinase inhibitor developed for the treatment of advanced non-small cell lung cancer (NSCLC) [1]. Osimertinib is particularly effective in NSCLC harboring EGFR mutations [2]. The most commonly reported adverse effects associated with osimertinib treatment include cardiotoxicity, pneumonia, ocular disorders, and dermatological reactions. Cardiovascular adverse events include QT-interval prolongation, cardiomyopathy, and heart failure, which may necessitate treatment discontinuation in some patients [3,4,5]. Other reported adverse events include thrombocytopenia and paronychia, while serious events such as cerebral infarction, hepatic dysfunction, and venous thromboembolism have also been documented [3]. The mechanisms underlying osimertinib-associated cardiotoxicity have not yet been fully elucidated. However, recent studies have demonstrated that mitochondrial calcium (Ca^2+^) overload plays a critical role in the development of osimertinib-associated cardiac dysfunction and myocardial injury [6]. In addition to causing marked structural and functional alterations in the heart, osimertinib has been reported to disrupt mitochondrial ultrastructure, reduce adenosine triphosphate (ATP) synthase activity, and consequently suppress ATP production [7]. Taken together, these findings from the literature provide a rationale for investigating whether exogenous ATP administration may modify cardiac injury associated with osimertinib treatment.

ATP is a nucleoside triphosphate composed of adenine, a ribose sugar, and three phosphate groups [8]. In the present study, we investigated its potential protective effect against osimertinib-associated cardiotoxicity. Previous studies have shown that ATP is required for glutathione synthesis, thereby supporting cellular antioxidant defense against ROS-mediated oxidative stress [9]. Furthermore, reduced intracellular ATP levels have been associated with disturbances in Ca^2+^ homeostasis [10,11]. Altered Ca^2+^ homeostasis may contribute to increased ROS production, while severe ATP depletion has been associated with loss of cellular viability [11]. In addition, Ca^2+^ dysregulation may promote phospholipase A_2_ activation, thereby enhancing arachidonic acid metabolism and cyclooxygenase (COX)-mediated inflammatory signaling [12]. Excessive ROS production, in turn, can induce oxidative damage to DNA, proteins, and lipids. One of the most widely used and reliable markers of oxidative DNA damage is 8-hydroxy-2′-deoxyguanosine (8-OHdG), which is generated as a result of DNA oxidation [13]. Taken together, these findings suggest that ATP may exert a protective effect against potential osimertinib-associated cardiotoxicity. However, to the best of our knowledge, no previous studies have specifically investigated the effects of ATP against osimertinib-associated cardiotoxicity. Therefore, the present study aimed to investigate whether ATP administration attenuates biochemical and histopathological alterations associated with osimertinib-induced myocardial injury in rats.

## 2. Materials and Methods

### 2.1. Animals

A total of 24 male Wistar albino rats weighing 275–288 g were included in this experimental study. The animals were obtained from the Medical Experimental Application and Research Center of Erzincan Binali Yıldırım University and randomly allocated into four groups, with six rats in each group (*n* = 6). No formal a priori sample size calculation was performed. The sample size was determined based on previous experimental studies employing comparable animal models and experimental endpoints, while also considering the Reduction principle of the 3Rs to minimize the number of animals used. Throughout the experimental period, the rats were housed under standard laboratory conditions at an ambient temperature of 22 ± 2 °C with a 12-h light/12-h dark cycle. Standard pellet chow and water were provided ad libitum. All experimental procedures were approved by the Local Animal Experiments Ethics Committee of Erzincan Binali Yıldırım University (Approval No. 2026/06, 25 June 2026). The study was designed and conducted in accordance with Directive 2010/63/EU of the European Parliament and the ARRIVE 2.0 guidelines [14].

### 2.2. Chemicals

Osimertinib (Tagrisso^®^, 80 mg tablets; AstraZeneca, Cambridge, UK) was obtained from AstraZeneca (Istanbul, Türkiye), thiopental sodium from İ.E. Ulagay (Istanbul, Türkiye), and ATP from Zdorove Narodu (Kyiv, Ukraine).

### 2.3. Experimental Groups

Following acclimatization, the animals were randomly allocated to four experimental groups (*n* = 6 per group) using a random-number table: vehicle-treated healthy control group (HG), an osimertinib-only group (OSM), a group receiving osimertinib in combination with ATP at a dose of 5 mg/kg (AOSM-5), and a group receiving osimertinib in combination with ATP at a dose of 10 mg/kg (AOSM-10).

### 2.4. Experimental Procedure

The experimental protocol was continued for three weeks. During this period, animals in the AOSM-5 (*n* = 6) and AOSM-10 (*n* = 6) groups received ATP intraperitoneally (i.p.) at doses of 5 and 10 mg/kg, respectively, once daily. The 5 mg/kg dose was selected based on previous experimental evidence demonstrating protective effects of intraperitoneally administered ATP at this dose in rats [15], whereas the 10 mg/kg dose was included as a higher dose to investigate the potential dose-dependent protective effects of ATP. Rats in the healthy control (HG, *n* = 6) and osimertinib control (OSM, *n* = 6) groups received distilled water, used as the vehicle for ATP, via the same route. One hour after ATP or distilled water administration, rats in the OSM, AOSM-5, and AOSM-10 groups received osimertinib (Tagrisso^®^, AstraZeneca) at a dose of 50 mg/kg by oral gavage once daily for three weeks. Osimertinib tablets were crushed into a fine powder and freshly suspended in distilled water before each administration. The resulting suspension was thoroughly mixed immediately before each gavage administration to minimize sedimentation and ensure uniform drug distribution. The resulting suspension was administered at a volume of 1 mL/kg. To ensure identical handling conditions, rats in the HG group received an equivalent volume of distilled water (1 mL/kg) by oral gavage once daily for three weeks. The osimertinib dose and treatment duration were selected based on a previous experimental cardiotoxicity study in which osimertinib was administered orally to mice at doses of 25 or 50 mg/kg for 21 consecutive days [7]. Twenty-four hours after the final osimertinib administration (corresponding to 25 h after the final ATP administration), blood samples were collected from the tail vein for the determination of troponin-I (TPI) levels. The animals were then anesthetized with thiopental sodium (50 mg/kg) and sacrificed, and the heart tissues were rapidly excised. ATP, malondialdehyde (MDA) and total glutathione (tGSH) levels, superoxide dismutase (SOD), and catalase (CAT) activities were measured in the cardiac tissues. In addition, cardiac tissues were subjected to histopathological examination, and the expression of COX-1, COX-2, and 8-OHdG was evaluated using immunohistochemical methods. Finally, the biochemical, histopathological, and immunohistochemical findings obtained from all groups were compared and evaluated.

### 2.5. Biochemical Analyses

#### 2.5.1. Preparation of Samples for Biochemical Analyses

Approximately 0.2 g of cardiac tissue from each animal was collected and thoroughly washed with 0.9% (*w*/*v*) NaCl to remove residual blood. The tissue samples were then homogenized on ice in 2 mL of ice-cold phosphate-buffered saline (PBS; pH 7.4), corresponding to a tissue-to-buffer ratio of 1:10 (*w*/*v*), using a high-speed tissue homogenizer (T 18 digital ULTRA-TURRAX^®^ (IKA-Werke GmbH & Co. KG, Staufen, Germany)). The homogenates were centrifuged at 13,000× *g* for 20 min at 4 °C, and the resulting supernatants were carefully collected and stored at −80 °C until biochemical analyses.

#### 2.5.2. Determination of Tissue ATP Levels

Cardiac tissue ATP levels were measured using a commercially available rat-specific enzyme-linked immunosorbent assay (ELISA) kit (catalog no. SL1512RA; lot no. 20260201; Sunlong Biotech Co., Ltd., Shanghai, China), in accordance with the manufacturer’s instructions. Heart tissue homogenates prepared as described above were used for the assay. ATP concentrations were determined from the standard curve generated according to the kit protocol and normalized to the total protein content of each tissue sample. The results were expressed as pg/mg protein. Since ATP is a low-molecular-weight metabolite, antibody-based quantification may be susceptible to non-specific background and cross-reactivity; therefore, the ATP measurements obtained with this assay were interpreted primarily for relative comparisons among experimental groups rather than as absolute reference measurements of myocardial ATP content.

#### 2.5.3. Determination of Tissue MDA, tGSH, SOD, and CAT Levels

Cardiac tissue levels of MDA and tGSH and the activities of SOD and CAT were determined using rat-specific commercially available assay kits supplied by YL Biont Co., Ltd. (Shanghai, China), according to the manufacturer’s instructions. The corresponding catalog numbers were YLA0029Ra for MDA, YLA0121Ra for tGSH, YLA0115Ra for SOD, and YLA0123Ra for CAT.

#### 2.5.4. Determination of Total Protein Content

Total protein concentrations in the cardiac tissue homogenates were determined using the Bradford method [16], with bovine serum albumin as the standard. Absorbance was measured at 595 nm using a Beckman Coulter DU 730 UV/Vis Life Science Spectrophotometer (Beckman Coulter Inc., Brea, CA, USA).

#### 2.5.5. Plasma TPI Measurement

TPI levels in plasma samples obtained from the animals were determined using the VIDAS^®^ TPI Ultra kit (catalog no. 415386-415386-30; bioMérieux, Marcy-l’Étoile, France), based on the Enzyme-Linked Fluorescent Assay (ELFA) method. The entire analytical procedure was performed automatically on the VIDAS^®^ analyzer using the ready-to-use reagents supplied with the kit, in accordance with the manufacturer’s instructions.

### 2.6. Histopathological Evaluation

After necropsy, heart specimens were immediately immersed in 10% neutral-buffered formalin for fixation. The fixed tissues were processed using standard histological procedures, including dehydration through graded alcohols, clearing in xylene, and paraffin embedding. Paraffin-embedded tissue sections (5 μm) were prepared, mounted on poly-l-lysine-coated slides, and stained with hematoxylin and eosin (H&E). All tissue sections were independently evaluated by two pathologists who were blinded to the experimental groups. For each animal, six randomly selected, non-overlapping microscopic fields were examined using a ×40 objective lens. The mean score of the six evaluated fields was used as the animal-level score for each histopathological parameter for statistical analysis. Interstitial hemorrhage and mononuclear inflammatory cell infiltration were evaluated using a semiquantitative scoring system according to lesion severity: 0, absent; 1, mild; 2, moderate; and 3, strong.

### 2.7. Immunohistochemical Method

Paraffin-embedded cardiac tissue sections (5 μm thick) were mounted on poly-l-lysine-coated slides, deparaffinized in xylene, and rehydrated through a graded series of alcohol. Following washing with phosphate-buffered saline (PBS), endogenous peroxidase activity was blocked by incubating the sections with 3% H_2_O_2_ for 10 min. For antigen retrieval, the sections were treated with antigen retrieval solution at 500 W for two consecutive 5-min cycles. After washing with PBS, the sections were incubated overnight at 4 °C with primary antibodies against COX-1 (Santa Cruz Biotechnology, Dallas, TX, USA, Cat. No. sc-19998), COX-2 (BT-Lab, Cat. No. BT-AP08027) and 8-OHdG (Bioss, Cat. No. bs-1278R), each diluted 1:200. Immunodetection was subsequently performed using the Large Volume Detection System: anti-Polyvalent, HRP (Thermo Fisher Scientific, Waltham, MA, USA, Cat. No. TP-125-HL) according to the manufacturer’s instructions. Immunoreactivity was visualized using 3,3′-diaminobenzidine (DAB) as the chromogen. The sections were then counterstained with Mayer’s hematoxylin, mounted with Entellan, and examined under a light microscope. Immunopositivity for COX-1, COX-2, and 8-OHdG was evaluated semiquantitatively according to staining intensity using a five-point scoring system: absent (0), mild (1), moderate (2), strong (3), and very strong (4). Six animals per group were evaluated. For each animal, three non-consecutive tissue sections were examined, and six randomly selected non-overlapping microscopic fields were evaluated using a ×40 objective lens by two investigators blinded to the experimental groups. The mean score of the evaluated fields was used as the animal-level score for each immunohistochemical marker for statistical analysis. In addition to semiquantitative scoring, quantitative analysis of immunohistochemical staining was performed using ImageJ software (version 1.54p, National Institutes of Health (NIH), Bethesda, MD, USA). Six randomly selected non-overlapping fields were analyzed for each animal. The intensity of DAB immunopositivity for COX-1, COX-2, and 8-OHdG was quantified by optical density (OD) analysis within predefined regions of interest (ROIs). All images were acquired using identical microscope, magnification, illumination, and camera settings. DAB-associated optical density values were calculated from RGB images using ImageJ. Background intensity was determined from unstained tissue areas within each image and used for background correction. Corrected OD values were used as quantitative measures of immunohistochemical staining intensity, and all images were analyzed under identical conditions. For each animal, the OD values obtained from the six analyzed fields were averaged to obtain a single animal-level value, and these animal-level mean values (*n* = 6 animals per group) were used for statistical analysis.

### 2.8. Statistical Analysis

Biochemical and quantitative immunohistochemical data were expressed as mean ± standard deviation (SD). Normality was assessed using the Shapiro–Wilk test, and homogeneity of variances was evaluated using Levene’s test. Differences among the HG, OSM, AOSM-5, and AOSM-10 groups for biochemical parameters and quantitative optical density (OD) data obtained from immunohistochemical image analysis were analyzed using one-way analysis of variance (ANOVA) when the assumptions of normality and homogeneity of variance were satisfied. When the overall ANOVA indicated a statistically significant difference, pairwise comparisons between groups were performed using Tukey’s honestly significant difference (Tukey HSD) post-hoc test. Semiquantitative histopathological and immunohistochemical scores were analyzed using non-parametric statistical methods. Differences among the groups were evaluated using the Kruskal–Wallis test, followed by the Mann–Whitney U test for pairwise comparisons when a significant overall difference was detected. A *p* value < 0.05 was considered statistically significant.

## 3. Results

### 3.1. Biochemical Findings

#### 3.1.1. Cardiac Tissue ATP Levels

Analysis of cardiac tissue ATP levels showed a significant decrease in the OSM group compared with the HG group (*p* < 0.001). ATP levels in the AOSM-5 group were significantly higher than those in the OSM group (*p* < 0.001) but remained significantly lower than those in the HG group (*p* < 0.001). The AOSM-10 group exhibited significantly higher ATP levels than both the OSM and AOSM-5 groups (*p* < 0.001 for both comparisons). Although ATP levels in the AOSM-10 group approached those observed in the HG group, they remained significantly lower (*p* < 0.001) (Figure 1, Table 1).

#### 3.1.2. Cardiac Tissue MDA Analysis Results

Cardiac tissue MDA levels were significantly increased in the OSM group compared with the HG group (*p* < 0.001). Treatment with both AOSM-5 and AOSM-10 significantly reduced MDA levels compared with the OSM group (*p* < 0.001). MDA levels were significantly lower in the AOSM-10 group than in the AOSM-5 group (*p* < 0.001), indicating a greater protective effect at the higher dose. However, MDA levels in the AOSM-10 group remained significantly higher than those in the HG group (Figure 2, Table 1).

#### 3.1.3. Cardiac Tissue tGSH Analysis Results

Cardiac tissue tGSH levels were significantly decreased in the OSM group compared with the HG group (*p* < 0.001). Both AOSM-5 and AOSM-10 significantly increased tGSH levels compared with the OSM group (*p* < 0.001). The increase was significantly greater in the AOSM-10 group than in the AOSM-5 group (*p* < 0.001), although tGSH levels remained significantly lower than those in the HG group (Figure 2, Table 1).

#### 3.1.4. Cardiac Tissue SOD Analysis Results

SOD activity was significantly reduced in the OSM group compared with the HG group (*p* < 0.001). Treatment with AOSM-5 and AOSM-10 significantly increased SOD activity compared with the OSM group (*p* < 0.001). SOD activity was significantly higher in the AOSM-10 group than in the AOSM-5 group (*p* < 0.001), but remained significantly lower than that in the HG group (Figure 2, Table 1).

#### 3.1.5. Cardiac Tissue CAT Analysis Results

CAT activity was significantly decreased in the OSM group compared with the HG group (*p* < 0.001). Both AOSM-5 and AOSM-10 significantly increased CAT activity compared with the OSM group (*p* < 0.001). CAT activity was significantly higher in the AOSM-10 group than in the AOSM-5 group (*p* < 0.001). Although AOSM-10 markedly restored CAT activity toward the HG level, a statistically significant difference remained between the HG and AOSM-10 groups (Figure 2, Table 1).

#### 3.1.6. Plasma TPI Analysis Results

When the groups were compared in terms of plasma TPI levels, TPI levels were significantly increased in the OSM group compared with the HG group (*p* < 0.001). TPI levels in the AOSM-5 group were significantly lower than those in the OSM group (*p* < 0.001), but remained significantly higher than those in the HG group (*p* < 0.001). In the AOSM-10 group, TPI levels were significantly decreased compared with both the OSM and AOSM-5 groups (*p* < 0.001 for both comparisons). Furthermore, TPI levels in the AOSM-10 group approached those observed in the HG group, with no statistically significant difference between the two groups (*p* > 0.05) (Figure 3, Table 1).

### 3.2. Histopathological Findings

Histopathological examination revealed significant differences among the experimental groups in terms of hemorrhage and mononuclear cell infiltration (Table 2, *p* < 0.001). Cardiac tissues from the HG group exhibited preserved myocardial architecture without detectable histopathological alterations (Figure 4A). In contrast, the OSM group showed marked mononuclear cell infiltration within the interstitial areas accompanied by mild hemorrhage (Figure 4B). ATP administration significantly reduced hemorrhage at both doses. Mononuclear cell infiltration was less pronounced in the AOSM-5 group, although it did not differ significantly from that in the OSM group, whereas a significant reduction was observed in the AOSM-10 group compared with the OSM group. Accordingly, moderate mononuclear cell infiltration was observed in the AOSM-5 group (Figure 4C), while only mild infiltration was detected in the AOSM-10 group (Figure 4D).

### 3.3. Immunohistochemical Findings

Quantitative immunohistochemical analysis revealed marked differences in COX-1, COX-2, and 8-OHdG staining among the experimental groups (Table 3). ImageJ-based optical density (OD) analysis showed a marked reduction in COX-1 immunoreactivity in the OSM group compared with the HG group. ATP co-administration increased COX-1 immunoreactivity, with a greater increase observed in the AOSM-10 group than in the AOSM-5 group. In contrast, COX-2 and 8-OHdG immunoreactivity increased markedly in the OSM group compared with the HG group. ATP co-administration reduced the OD values of both COX-2 and 8-OHdG, with lower values observed in the AOSM-10 group than in the AOSM-5 group. Representative immunohistochemical images were consistent with the quantitative findings (Figure 5).

## 4. Discussion

In this study, the effects of ATP against the potential cardiotoxicity of osimertinib in rats were investigated. In addition, the relationship between cardiac tissue ATP levels and the potential cardiotoxicity of osimertinib was evaluated. Our biochemical findings demonstrated that osimertinib significantly reduced the measured ATP levels in cardiac tissue.

The cardiotoxic effects of osimertinib have attracted increasing attention in recent years. Clinical studies and pharmacovigilance analyses have reported cardiomyopathy, heart failure, QT-interval prolongation, and various cardiac events associated with osimertinib use [4,5]. However, the molecular mechanisms underlying this cardiotoxicity have not yet been fully elucidated. Nevertheless, recent studies have shown that osimertinib causes structural abnormalities in cardiac mitochondria, suppresses ATP synthase activity, and reduces cellular energy production [7]. Deng et al. further reported that osimertinib disrupts mitochondria–endoplasmic reticulum interactions, thereby triggering mitochondrial calcium overload, oxidative stress, and necroptotic cell death [6].

Previous studies suggest that ATP depletion, Ca^2+^ dysregulation, mitochondrial dysfunction, and oxidative stress may represent interconnected processes involved in myocardial injury [10,11]. However, these mechanisms were not directly evaluated in the present study and should therefore be regarded as literature-based mechanistic inferences, rather than mechanisms demonstrated by our findings. Consistent with the presence of oxidative stress, MDA, a toxic product of lipid peroxidation (LPO) [17], was significantly increased in the osimertinib-only group, whereas tGSH levels and the activities of the antioxidant enzymes SOD and CAT [17,18] were significantly decreased. Because cytosolic and mitochondrial Ca^2+^ levels, mitochondrial membrane potential, mitochondria–ER interactions, and necroptosis-related markers were not assessed, the contribution of these processes to osimertinib-associated myocardial injury cannot be established based on the present findings. Further studies directly evaluating these pathways are required to clarify their potential contribution.

Although the absence of an ATP-alone group prevents definitive differentiation between the independent cardiovascular effects of ATP and its effects under conditions of osimertinib exposure, the overall pattern of the present findings supports the interpretation that ATP attenuates osimertinib-associated myocardial injury. Osimertinib produced a consistent pattern of injury characterized by increased lipid peroxidation and plasma troponin I levels, impaired antioxidant defenses, oxidative DNA damage, altered COX-1/COX-2 immunoreactivity, and histopathological evidence of myocardial injury. ATP co-administration attenuated these alterations across multiple independent endpoints, generally in a dose-dependent manner. These findings are consistent with previous studies reporting a close relationship between ATP levels and oxidative stress [11]. Furthermore, from a pharmacological perspective, the observed effects should not be considered to result from direct replenishment of the intracellular ATP pool. Extracellular ATP can act through P2 purinergic receptors and is rapidly metabolized by ectonucleotidases to adenosine, which can exert biological effects through P1 receptors [19]. Such purinergic signaling may modulate oxidative and inflammatory responses and limit cellular and mitochondrial stress, thereby indirectly contributing to the preservation of cellular homeostasis and the endogenous capacity of cardiomyocytes to maintain ATP production under osimertinib-induced stress. Thus, the observed increase in measured myocardial ATP levels may reflect the preservation of endogenous energy metabolism rather than direct cellular uptake of the administered ATP. Since purinergic receptor activation, adenosine levels, mitochondrial function, and ATP synthesis were not directly evaluated in the present study, this mechanistic interpretation remains hypothetical. Thus, the concordant improvements observed across biochemical, histopathological, and immunohistochemical markers are consistent with attenuation of osimertinib-associated myocardial injury. Nevertheless, because ATP itself can exert cardiovascular effects and an ATP-alone group was not included, the present study cannot definitively establish that these effects represent a specific antagonistic action against osimertinib. Furthermore, ATP uptake, adenosine levels, and purinergic signaling were not directly evaluated; therefore, the precise mechanism underlying the observed attenuation of myocardial injury remains unclear and should be investigated in future studies incorporating ATP-alone control groups.

To evaluate the cardiotoxicity of osimertinib, plasma TPI levels were measured in the experimental animals. Our findings demonstrated that osimertinib increased plasma TPI levels and that ATP administration attenuated this increase in a dose-dependent manner. TPI is a highly sensitive and specific biomarker of myocardial injury, and its release into the circulation may occur in response to cardiomyocyte damage associated with ischemia, inflammation, oxidative stress, and cardiotoxic agents [20,21]. Markedly elevated plasma troponin I levels have also been reported in cases of osimertinib-associated eosinophilic myocarditis [22], supporting the possibility that the increase in troponin observed in our study may be associated with cardiac injury.

One of the major consequences of oxidative stress is the activation of the inflammatory response [23]. Increased ROS levels and disturbances in Ca^2+^ homeostasis are associated with phospholipase A_2_ activation, which may enhance arachidonic acid metabolism and COX-mediated inflammatory signaling [12]. In our study, COX-1 expression was decreased, whereas COX-2 expression was markedly increased in the osimertinib group. Prostaglandins produced through COX-1 primarily contribute to the regulation of physiological homeostatic functions, whereas prostaglandins generated through COX-2 are predominantly involved in mediating inflammation [24].

The restoration of COX-1 expression and suppression of COX-2 expression following ATP treatment suggest that ATP may attenuate inflammatory alterations. This protective effect may be associated with the attenuation of oxidative stress by ATP. Although osimertinib has been reported to reduce COX-2 expression in some tumor models [25], the decrease in COX-1 and increase in COX-2 observed in our study may be related to mitochondrial dysfunction, oxidative stress, and inflammatory processes associated with reduced ATP levels.

Excessive ROS production causes oxidative damage not only to lipids and proteins but also to DNA. 8-OHdG, one of the most reliable markers of DNA oxidation, reflects the extent of oxidative DNA damage [13]. The marked increase in 8-OHdG immunopositivity in the osimertinib-treated group indicates increased oxidative DNA damage in cardiac tissue following osimertinib administration. The reduction in 8-OHdG expression following ATP administration suggests that ATP may protect DNA against oxidative damage. The increase in 8-OHdG accompanying the reduction in ATP levels observed in our study is consistent with previous reports linking impaired cellular energy production and mitochondrial dysfunction with oxidative DNA damage [26].

In the cardiac tissue of the osimertinib-treated group, mild hemorrhage and severe mononuclear cell infiltration in the interstitial areas were observed. These histopathological alterations were less pronounced in the group treated with ATP at a dose of 10 mg/kg than in the group treated with 5 mg/kg ATP. The marked mononuclear cell infiltration and mild hemorrhage observed in the osimertinib group suggest that osimertinib may induce an inflammatory response and cellular injury in cardiac tissue. These findings are consistent with previous reports indicating that osimertinib-associated cardiotoxicity involves an inflammatory component [22,27].

This study has several limitations. First, because the study was conducted using an experimental rat model, the direct generalizability of the findings to human cardiac physiology is limited. Second, the mechanisms underlying cardiotoxicity were evaluated primarily using biochemical, immunohistochemical, and histopathological parameters, while more detailed assessments of mitochondrial function were not performed. Third, specific cytokine profiles, including TNF-α, IL-6, and IL-1β, were not analyzed to further characterize the inflammatory response. Furthermore, functional cardiac assessments, including electrocardiography and echocardiography, were not performed. Therefore, the biochemical and histopathological improvements observed with ATP cannot be directly generalized as evidence of functional cardioprotection. Another limitation is the absence of ATP-only control groups, which precluded assessment of the independent effects of ATP on cardiac biochemical, histopathological, and immunohistochemical parameters in healthy animals. In the present study, ATP measurements were performed using the same commercially available kit, with standardized sample-preparation procedure, tissue-to-buffer ratio, assay conditions, and analytical workflow for all experimental groups. Therefore, the ATP levels were used primarily for relative comparisons among experimental groups rather than as an absolute reference measurement of myocardial ATP content. Importantly, our interpretation of these findings is based on the direction and magnitude of group differences under identical analytical conditions and not on the assumption that the immunoassay provides the analytical specificity achievable with chromatographic or luciferase-based methods. In addition, because cardiac tissues were homogenized in ice-cold PBS without an acid-based protein precipitation step, such as perchloric acid (PCA) or trichloroacetic acid (TCA) extraction, ATPase activity may not have been immediately and completely inhibited during sample processing. Therefore, some degree of ATP degradation during sample preparation may have occurred. Accordingly, the ATP values obtained using the commercial immunoassay and expressed as pg/mg protein should not be interpreted as absolute physiological myocardial ATP concentrations or directly compared with ATP concentrations reported using validated metabolic extraction procedures combined with luciferase-based or chromatographic methods. Therefore, these methodological considerations should be taken into account when interpreting the ATP findings of the present study. Also, no formal a priori sample size calculation was performed; the sample size was determined based on previous experimental studies employing comparable animal models and experimental endpoints, while also considering the Reduction principle of the 3Rs to minimize the number of animals used. In addition, only male rats were included in the present study; therefore, potential sex-related differences in osimertinib-associated cardiac injury and the response to ATP could not be evaluated. Our study was conducted in healthy, non-tumor-bearing animals, which did not allow us to determine whether ATP administration affects the antitumor efficacy of osimertinib. To clarify this important issue, future studies using tumor-bearing models and/or similar studies in EGFR-mutant cancer cells and cardiomyocytes are required. Finally, the long-term effects of ATP and its pharmacodynamic properties across different dose ranges were not investigated; therefore, no definitive conclusions can be drawn regarding the optimal dose and safety profile for potential clinical application.

## 5. Conclusions

In this study, osimertinib administration was associated with marked cardiac injury characterized by reduced measured cardiac ATP levels, increased oxidative stress and oxidative DNA damage, impaired antioxidant defenses, elevated TPI levels, and histopathological evidence of inflammatory cell infiltration. Exogenous ATP administration attenuated these alterations, with generally more pronounced protective effects observed at the 10 mg/kg dose. These findings indicate that ATP co-administration attenuated the biochemical, histopathological, and immunohistochemical alterations associated with osimertinib-induced myocardial injury in rats. These effects may be associated, at least in part, with attenuation of oxidative stress. However, further studies are required to clarify the underlying mechanisms, determine whether these tissue-level effects translate into functional cardiac benefits, and establish whether ATP administration influences the antitumor efficacy of osimertinib.

## Figures and Tables

**Figure 1:**
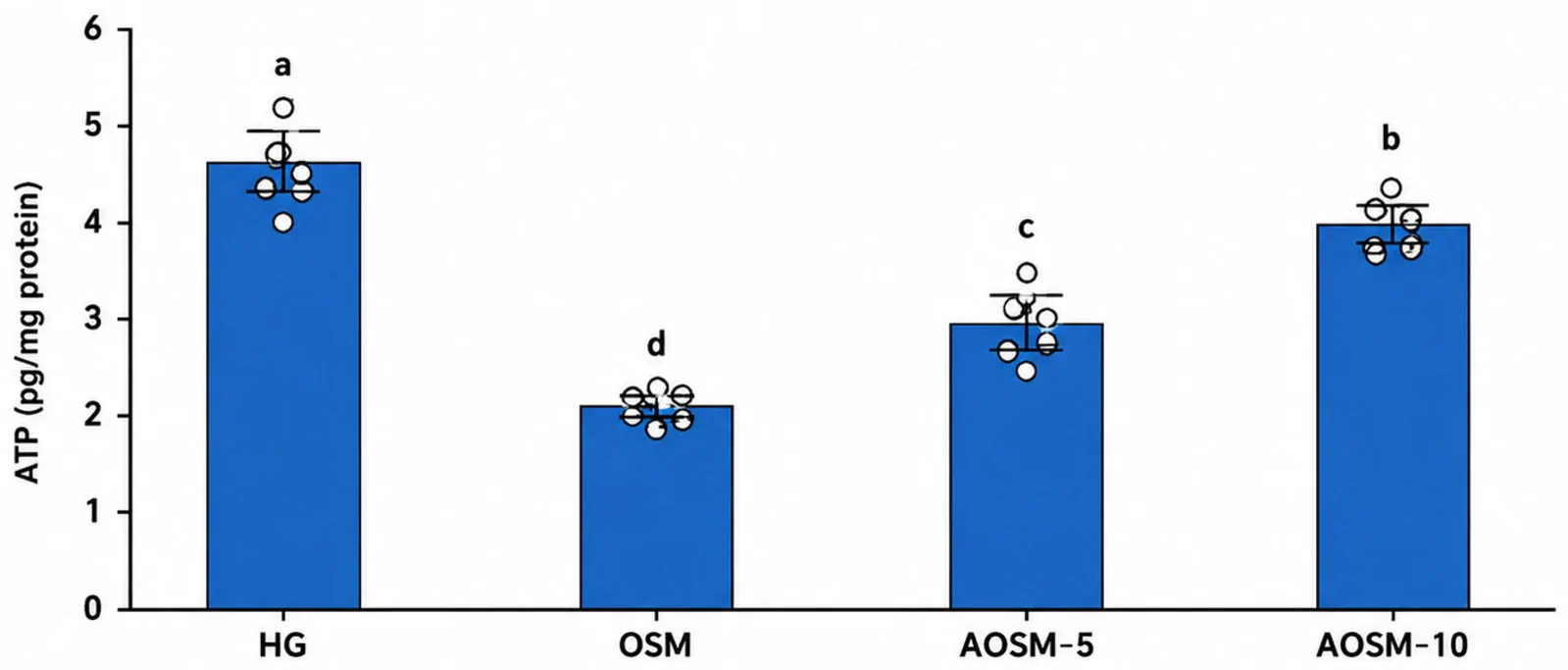
Cardiac tissue ATP levels in the experimental groups. Data are presented as mean ± SD (*n* = 6 per group), with individual data points shown. Statistical analysis was performed using one-way ANOVA followed by Tukey’s HSD post-hoc test. Different lowercase letters (a–d) indicate statistically significant differences between groups (*p* < 0.05). Abbreviations: HG, healthy group; OSM, osimertinib group; AOSM-5, ATP (5 mg/kg) + osimertinib group; AOSM-10, ATP (10 mg/kg) + osimertinib group.

**Figure 2:**
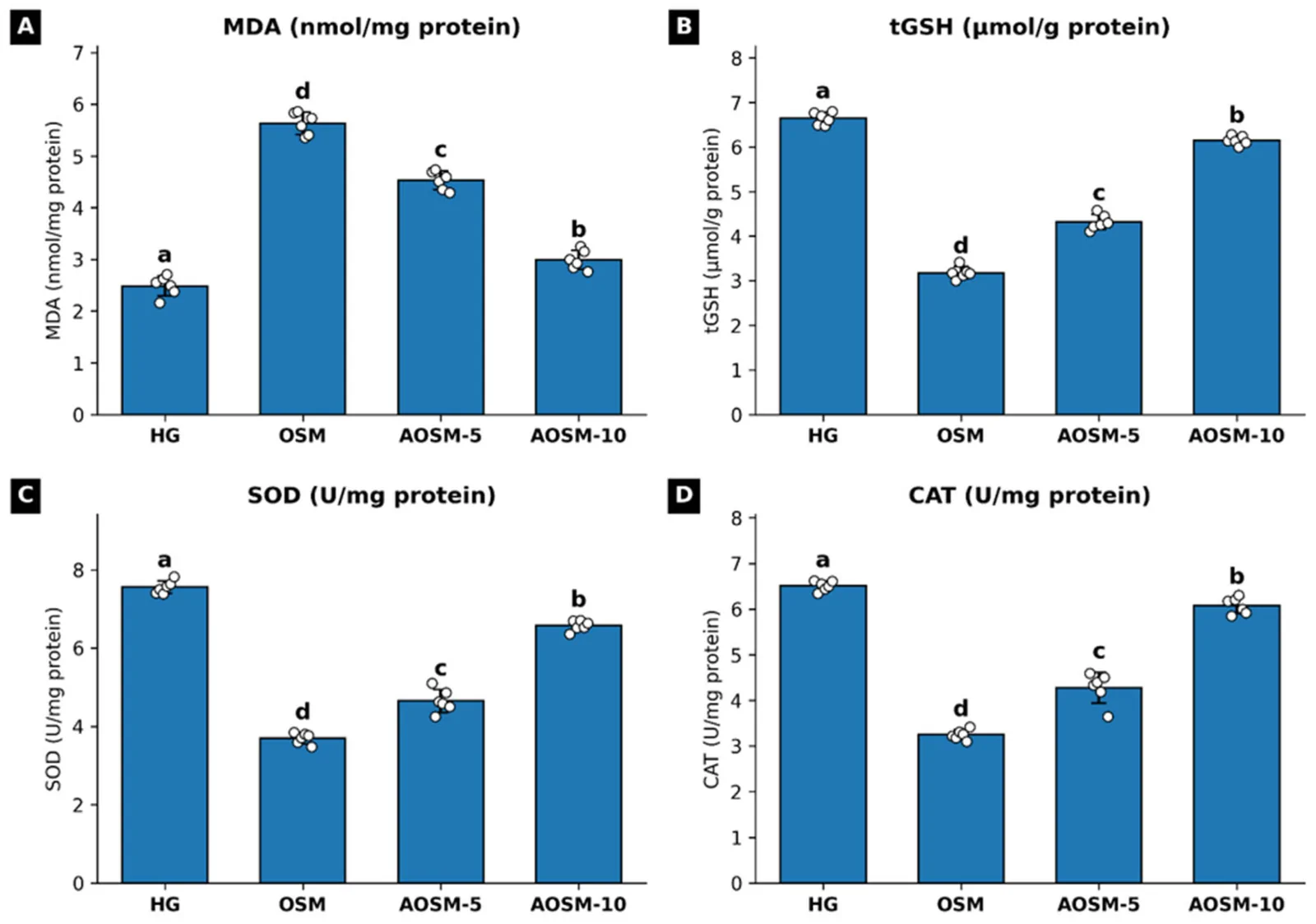
Effects of AOSM-5 and AOSM-10 treatments on oxidative stress and antioxidant parameters in cardiac tissue. (**A**) MDA levels, (**B**) tGSH levels, (**C**) SOD activity, and (**D**) CAT activity in the HG, OSM, AOSM-5, and AOSM-10 groups. Data are presented as mean ± SD (*n* = 6 per group), with individual data points shown. Statistical analysis was performed using one-way ANOVA followed by Tukey’s HSD post-hoc test. Different lowercase letters (a–d) indicate statistically significant differences between groups (*p* < 0.05). Abbreviations: HG, healthy group; OSM, osimertinib group; AOSM-5, ATP (5 mg/kg) + osimertinib group; AOSM-10, ATP (10 mg/kg) + osimertinib group.

**Figure 3:**
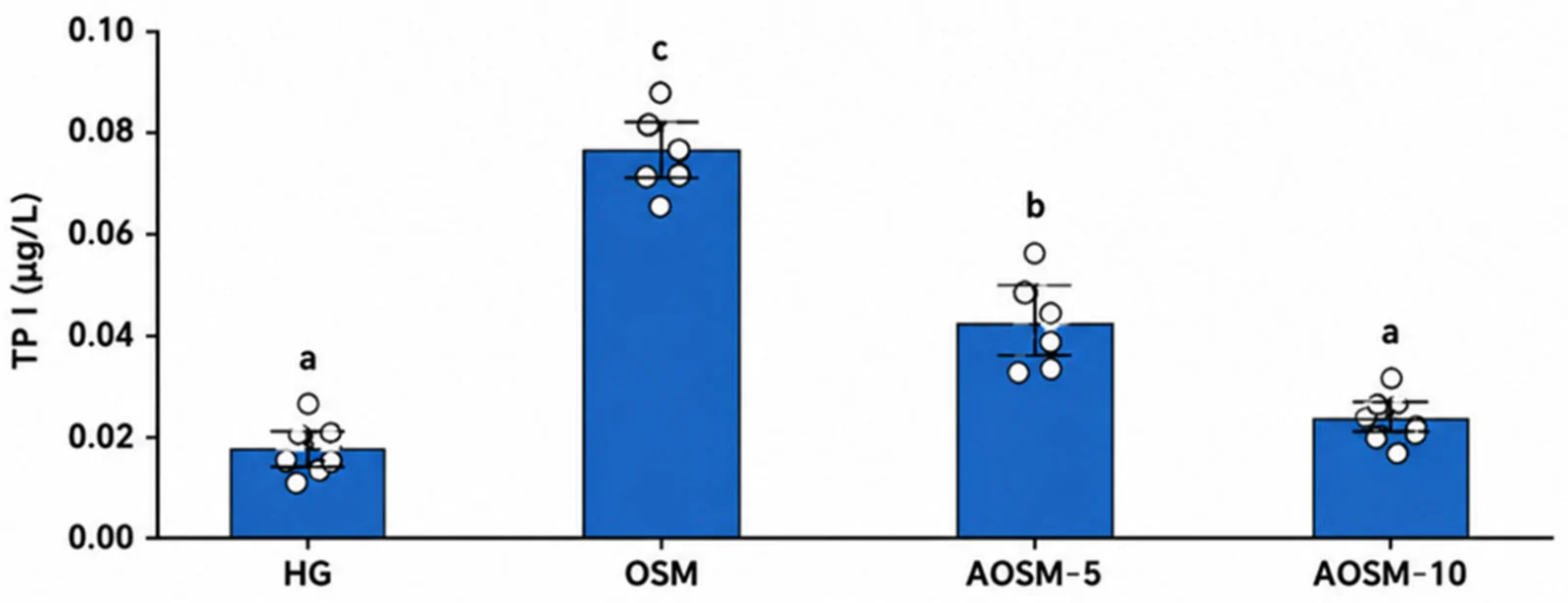
Plasma troponin I (TPI) levels in the experimental groups. Data are presented as mean ± SD (*n* = 6 per group), with individual data points shown. Statistical analysis was performed using one-way ANOVA followed by Tukey’s HSD post-hoc test. Groups sharing the same lowercase letter are not significantly different, whereas groups with different letters are significantly different (*p* < 0.05). Abbreviations: HG, healthy group; OSM, osimertinib group; AOSM-5, ATP (5 mg/kg) + osimertinib group; AOSM-10, ATP (10 mg/kg) + osimertinib group.

**Figure 4:**
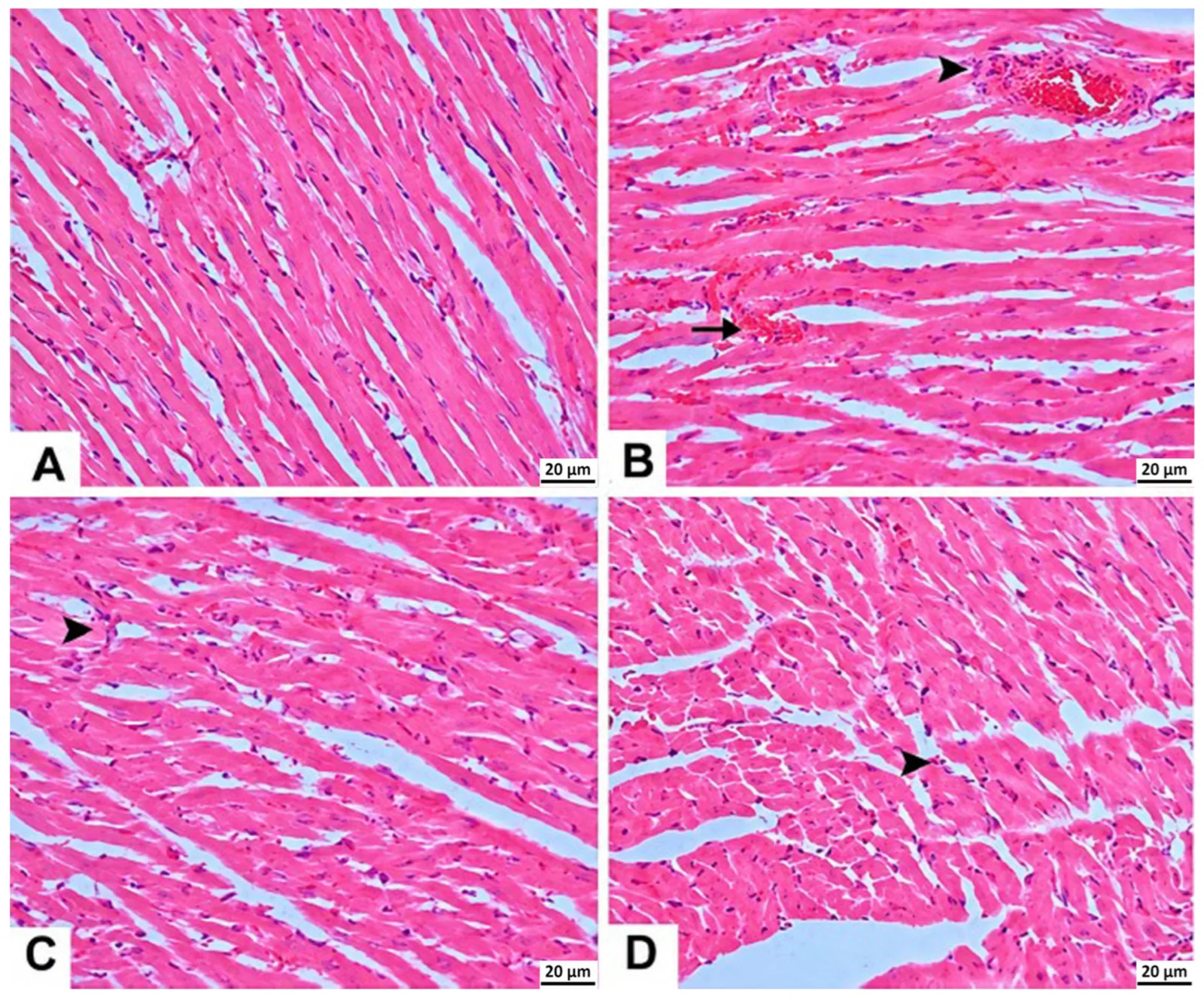
Representative histopathological images of cardiac tissue from the experimental groups. (**A**) HG group showing preserved myocardial architecture without apparent histopathological alterations. (**B**) OSM group showing mild interstitial hemorrhage with red blood cell extravasation (**→**) and severe mononuclear inflammatory cell infiltration (➤). (**C**) AOSM-5 group showing moderate mononuclear inflammatory cell infiltration (➤). (**D**) AOSM-10 group showing mild mononuclear inflammatory cell infiltration (➤). Hematoxylin and eosin (H&E) staining; objective magnification: ×40; scale bar: 20 μm. Abbreviations: HG, healthy group; OSM, osimertinib group; AOSM-5, ATP (5 mg/kg) + osimertinib group; AOSM-10, ATP (10 mg/kg) + osimertinib group.

**Figure 5:**
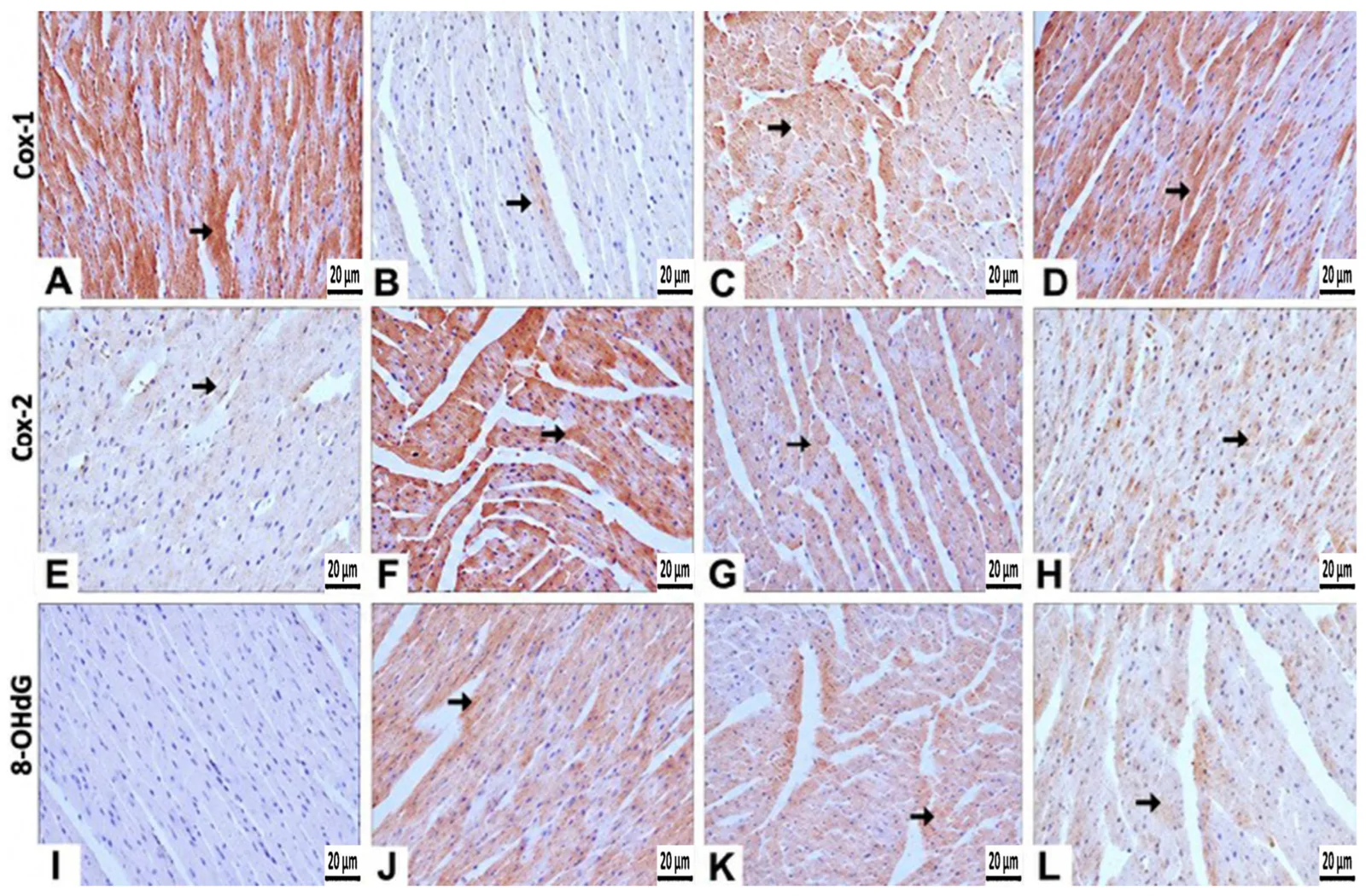

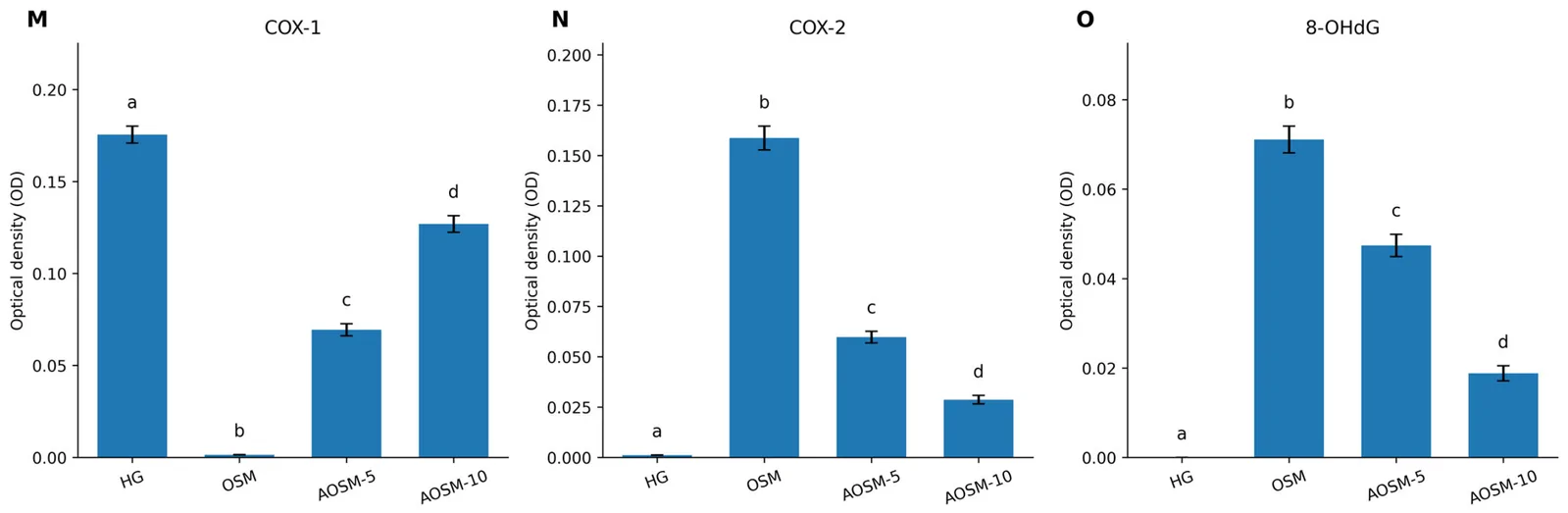
Representative immunohistochemical images and quantitative optical density analysis of COX-1, COX-2, and 8-OHdG staining in cardiac tissue from the experimental groups. COX-1 immunoreactivity: (**A**) HG, (**B**) OSM, (**C**) AOSM-5, and (**D**) AOSM-10. COX-2 immunoreactivity: (**E**) HG, (**F**) OSM, (**G**) AOSM-5, and (**H**) AOSM-10. 8-OHdG immunoreactivity: (**I**) HG, (**J**) OSM, (**K**) AOSM-5, and (**L**) AOSM-10. Arrows indicate representative areas of DAB-positive immunoreactivity. DAB immunohistochemical staining; objective magnification: ×40; scale bar: 20 μm. Quantitative optical density (OD) analysis was performed using ImageJ for COX-1 (**M**), COX-2 (**N**), and 8-OHdG (**O**). Quantitative data are presented as mean ± SD (*n* = 6 per group). Statistical comparisons were performed using one-way ANOVA followed by Tukey’s HSD post hoc test. Different letters indicate statistically significant differences between groups (*p* < 0.05). The corresponding numerical values are provided in Table 3.

**Table 1:** Cardiac Tissue MDA, tGSH, and ATP Levels, SOD and CAT Activities, and Plasma Troponin I Levels in the Experimental Groups.

Parameter	HG	OSM	AOSM-5	AOSM-10	ANOVA
MDA	2.49 ± 0.19 ^a^	5.63 ± 0.21 ^d^	4.53 ± 0.18 ^c^	2.99 ± 0.19 ^b^	F(3,20) = 326.86, *p* < 0.001
tGSH	6.64 ± 0.14 ^a^	3.18 ± 0.14 ^d^	4.32 ± 0.17 ^c^	6.15 ± 0.10 ^b^	F(3,20) = 819.50, *p* < 0.001
SOD	7.56 ± 0.16 ^a^	3.70 ± 0.14 ^d^	4.65 ± 0.29 ^c^	6.58 ± 0.13 ^b^	F(3,20) = 496.04, *p* < 0.001
CAT	6.51 ± 0.11 ^a^	3.25 ± 0.11 ^d^	4.28 ± 0.34 ^c^	6.08 ± 0.18 ^b^	F(3,20) = 330.24, *p* < 0.001
ATP	4.56 ± 0.13 ^a^	2.26 ± 0.08 ^d^	2.91 ± 0.11 ^c^	4.01 ± 0.09 ^b^	F(3,20) = 633.34, *p* < 0.001
TPI	0.0157 ± 0.0030 ^a^	0.0753 ± 0.0056 ^c^	0.0415 ± 0.0068 ^b^	0.0227 ± 0.0034 ^a^	F(3,20) = 173.95, *p* < 0.001

Data are presented as mean ± SD (*n* = 6 per group). Statistical analysis was performed using one-way ANOVA followed by Tukey’s HSD post-hoc test. Different superscript letters (a–d) within the same row indicate statistically significant differences between groups (*p* < 0.05). Abbreviations: HG, healthy group; OSM, osimertinib group; AOSM-5, ATP (5 mg/kg) + osimertinib group; AOSM-10, ATP (10 mg/kg) + osimertinib group.

**Table 2:** Histopathological findings in cardiac tissue among the experimental groups.

Groups	Hemorrhage Median (Min–Max)	Mononuclear Cell Infiltration Median (Min–Max)
HG	0 (0–0) ^a^	0 (0–1) ^a^
OSM	1 (1–2) ^b^	3 (2–3) ^b^
AOSM-5	0 (0–0) ^a^	2 (1–2) ^b^
AOSM-10	0 (0–0) ^a^	1 (0–1) ^c^
Kruskal–Wallis H	22.582	19.414
*p* value	<0.001	<0.001

Data are presented as median (min–max). Differences among groups were analyzed using the Kruskal–Wallis test, followed by pairwise Mann–Whitney U tests. Different superscript letters indicate statistically significant differences between groups (*p* < 0.05). Abbreviations: HG, healthy group; OSM, osimertinib group; AOSM-5, ATP (5 mg/kg) + osimertinib group; AOSM-10, ATP (10 mg/kg) + osimertinib group.

**Table 3:** Quantitative immunohistochemical analysis of COX-1, COX-2, and 8-OHdG expression in cardiac tissue.

Experimental Group	COX-1 (OD)	COX-2 (OD)	8-OHdG (OD)
HG	0.1755 ± 0.0046 ^a^	0.0011 ± 0.0001 ^a^	0.0000 ± 0.0000 ^a^
OSM	0.0014 ± 0.0001 ^b^	0.1587 ± 0.0059 ^b^	0.0711 ± 0.0030 ^b^
AOSM-5	0.0694 ± 0.0033 ^c^	0.0598 ± 0.0029 ^c^	0.0474 ± 0.0025 ^c^
AOSM-10	0.1269 ± 0.0045 ^d^	0.0287 ± 0.0021 ^d^	0.0188 ± 0.0017 ^d^

Data are presented as mean ± standard deviation (SD) of optical density (OD) values (*n* = 6 animals per group). OD values were calculated following background correction using the formula OD = log10 (Mean_background/Mean_target). Differences among groups were analyzed using one-way ANOVA followed by Tukey’s HSD post-hoc test. Different superscript letters (a–d) within the same column indicate statistically significant differences between groups (*p* < 0.05). Abbreviations: HG, healthy group; OSM, osimertinib group; AOSM-5, ATP (5 mg/kg) + osimertinib group; AOSM-10, ATP (10 mg/kg) + osimertinib group; OD, optical density; SD, standard deviation.

## Data Availability

The data generated and analyzed during the present study are available from the corresponding author upon reasonable request.

## Abbreviations

The following abbreviations are used in this manuscript:

EGFR	Epidermal growth factor receptor
NSCLC	Non-small cell lung cancer
ATP	Adenosine triphosphate
COX	Cyclooxygenase
8-OHdG	8-hydroxy-2′-deoxyguanosine
QT	QT interval
ROS	Reactive oxygen species
TPI	Troponin-I
MDA	Malondialdehyde
tGSH	Total glutathione
SOD	Superoxide dismutase
CAT	Catalase
H&E	Hematoxylin and eosin
ELISA	Enzyme-linked immunosorbent assay
PBS	Phosphate-buffered saline
ANOVA	One-way analysis of variance
PCA	Perchloric acid
TCA	Trichloroacetic acid
